# Living with dementia: increased level of caregiver stress in times of COVID-19

**DOI:** 10.1017/S1041610220001593

**Published:** 2020-07-30

**Authors:** Gabriela Cohen, María Julieta Russo, Jorge A. Campos, Ricardo F. Allegri

**Affiliations:** Fundación para la Lucha contra las Enfermedades Neurológicas de la Infancia, Memory and Ageing Center, Department of Cognitive Neurology, Neuropsychology and Neuropsychiatry (FLENI), Buenos Aires, Argentina

**Keywords:** Alzheimer’s disease (AD), dementia, outpatient

## Abstract

COVID-19 pandemic in Argentina has affected the care of older adults with dementia deeply. Our objective was to study how the obligatory social isolation affected stress caregiver and burden of care of family members of subjects living with dementia in the community after the initial 4 weeks of quarantine in our setting. We did a questionnaire survey among 80 family caregivers of persons with Alzheimer’s disease (AD) or related dementia collected on April 2020. We designed a visual analog scale to test the level of the burden of care. Characteristics of people with dementia and their caregivers were analyzed with descriptive (mean, standard deviation, frequency and percent) and inferential statistics (chi-square test). The sample included older adults (mean age: 80.51 ± 7.65) with different stages of dementia. Family was the primary provider of care in 65%. Overall, COVID-19 confinement increased stress caregiver independently of the dementia stage, but those caring for severe cases had more stress compared to milder forms of the disease. Other findings were that half of the subjects with dementia experienced increased anxiety and that most family members discontinued all sort of cognitive and physical therapies. Family members’ main concerns were for severe dementia cases, fear of absence of the paid caregiver during the epidemic, and for mild cases fear of spreading the disease while assisting patients with instrumental activities. A partnership between departments of public health, care workers and families must be planned to guarantee continuity of care during these unique COVID-19 times.

## Introduction

Coronavirus disease (COVID-19) pandemic has deeply affected the care that older adults with Alzheimer’s disease (AD) and related disorders received in Argentina. Even though circulation for family caregivers of subjects with dementia was one of the few exceptions allowed by the government (Ministerio de la Nación, 2020), we observed that most family members decided to stop visiting their relatives from fear of spreading the disease.

COVID-19 epidemic is causing a radical change in the model of dementia care. Before this pandemic, engaging in social activities, performing cognitive and physical activities, and having a productive daily routine have been the mainstay therapy (Austrom *et al.*, 2018). To relief caregiver stress, literature has shown that multicomponent strategies such as avoiding isolation, attending family and group support meetings, and sharing the burden of care with other family members were useful (Hughes *et al.*, 2014). Now, in times of COVID-19, we recommend the most strict social isolation, especially for older patients with dementia and other comorbidities who have the highest risk for severe COVID-19 disease and mortality (Emami *et al.*, 2020).

Previous quarantines in human history had a negative psychological impact on outcomes such as anger, depression, and loneliness in the general population (Brooks *et al.*, 2020), but the effects on the well-being and standard care of subjects with dementia living in the community are not well studied. The objective of our research was to study to what extend mandatory social isolation affected the stress and burden of care of family members caring for subjects with dementia after the initial 4 weeks of quarantine and to study the relationship between the severity of the dementia, measured with the Clinical Dementia Rating (CDR) (Hughes *et al*., 1982), and the impact of the negative effects of quarantine in our setting.

## Material and methods

This brief report is based on data of a questionnaire survey among 80 family caregivers of persons with AD or related dementia, collected during 2 days of April 2020 from the Aging and Memory Center at FLENI in Buenos Aires. All participants had a comprehensive diagnostic evaluation before the isolation period by a member of the team with experience in Cognitive Neurology.

We specifically designed for our study an online questionnaire with an accessible format to be completed by family members of persons with AD or related dementia. The questionnaire had simple and easy to answer questions. It was not intended to replace medical consultations, validate tests, or make accurate neuropsychiatric diagnoses. Items asked included demographic characteristics of both subjects with dementia and family members and problems of management, rehabilitation, and care that subjects experienced during the first 4 weeks of the coronavirus quarantine in our setting.

To test the presence of anxiety, we asked caregivers two simple questions: “Did your relative with dementia experienced anxiety before the pandemic?” and “Do your relative with dementia experienced anxiety during the pandemic?

To measure the amount of burnout and stress that a family caregiver experienced, we asked the following question: “How much stress related to the care of your family member with AD are you experiencing during this quarantine?” For this purpose, we designed a visual analog scale, with a continuous range from none to an extreme amount of stress. Based on the obtained score, the results were transformed into three categories: low, medium, and high level of burden.

To overcome some of the limitations imposed by conventional pretest–posttest self-report measures, a retrospective pretest–posttest design was utilized. We made this selection since a retrospective pretest–posttest design is a convenient and valid method for measuring self-reported change. This method has shown to reduce response shift bias with more accurate assessments of actual effect and provides comparison data in the absence of “pre” data (Nimon, 2014).

### Analyses

The characteristics of people with AD or related dementia and their caregivers were analyzed with descriptive (mean, standard deviation, frequency, and percent) and inferential statistics. To evaluate the association of dementia stage, caregiver burnout, and main clinical data, chi-square tests (dependent variable: dementia stages based on CDR score) were used. The main results were presented as percentages of studied variables to provide a straightforward summary statistic easily understood. Statistical analyses were performed using SPSS 21.0, with statistical significance set at *p* < .05.

### Ethics

A letter with an invitation to participate in the survey was mailed to family members explaining the objectives of the research study. Confidentiality of family members and subjects with AD were strictly preserved through all phases of the survey process and forever after. According to the Argentina law, no approval of a Medical Ethics Committee is needed for a survey research that does not involve any intervention.

## Results

Characteristics of participating caregivers and persons with AD or related dementia are reported in Table 1. Twenty-three family caregivers (28.8% of the total sample) had a relative in the initial stage of dementia (CDR = 1), 33 (41.3%) in an intermediate stage (CDR = 2), and 24 (30.0%) had a relative who have severe dementia (CDR = 3). The most frequent subtype of dementia was AD, followed by mixed AD, with 61.3% and 20.0%, respectively. The average age of the sample was 80.51 ± 7.65, and the average education was 13.95 ± 4.94. Women accounted for 62.5% of persons with dementia.

**Table 1.** Characteristics of participating caregivers and persons with Alzheimer’s disease or related dementia (in percentages)

Variables	CDR			*χ* ^2^	*p*
	1	2	3		
**Part I: Persons with Dementia**					
Age				2.996	.558
<45 years old	33.30%	11.10%	55.60%		
45–65 years old	23.80%	57.10%	19.00%		
65–85 years old	25.00%	37.50%	37.50%		
≥85 years old	0.00%	100.00%	0.00%		
Gender				0.718	.698
Male	23.30%	43.30%	33.40%		
Female	32.00%	40.00%	28.00%		
Diagnosis				10.455	.107
AD	28.60%	44.90%	26.50%		
Mixed AD	37.50%	43.80%	18.80%		
Vascular dementia	42.90%	28.60%	28.60%		
Others	0.00%	25.00%	75.00%		
Caregiver				3.497	.174
Paid caregiver	21.40%	35.70%	42.90%		
Family caregiver	32.70%	44.20%	23.10%		
**Part II: Family Caregivers**					
Age				7.514	.276
<45 years old	33.30%	11.10%	55.60%		
45–65 years old	23.80%	57.10%	19.00%		
65–85 years old	25.00%	37.50%	37.50%		
≥85 years old	0.00%	100.00%	0.00%		
Gender				.428	.372
Male	25.44%	41.66%	33.00%		
Female	26.00%	44.40%	29.60%		
Do not visit a family member with dementia during the COVID-19 pandemic				.917	.632
No	28.60%	44.90%	26.50%		
Yes	29.00%	35.50%	35.50%		
Do not allow services of paid caregivers during the COVID-19 pandemic				8.981	**.011**
No	11.10%	53.50%	35.60%		
Yes	44.40%	27.80%	27.80%		
Level of burden of the family caregiver during the COVID-19 pandemic				17.281	**.002**
Low burden	27.00%	21.60%	51.40%		
Medium burden	8.60%	34.30%	57.10%		
High burden	10.00%	37.10%	52.90%		
Questions about supporting someone with dementia during the coronavirus outbreak					
I’m worried that the paid caregivers who come in to help us might not be able to come.	15.10%	39.40%	45.50%	8.340	**.017**
I need to go outside to pick up supplies for my relative with dementia but I am worried that I might catch the virus.	56.60%	30.40%	13.00%	10.499	**.005**

*Notes*: This table represents valid percentage of responses on the questionnaire survey specifically designed for this study. The bold represents *p* < .05.

*Abbreviations*: CDR, Clinical Dementia Rating; AD, Alzheimer’s disease.

Of our sample, 43.8% of people with AD or related dementia experienced anxiety before the coronavirus pandemic without a statistical difference along the spectrum of severity of the disease based on CDR score (*χ* ^2^ (2) = .281, *p* = .869). Forty-eight percent of the sample reported an increased level of anxiety in the context of containment for COVID-19. Of these 39 patients, 12 needed an increase in antipsychotics doses and 7 needed an increase in benzodiazepines doses.

Before the pandemic, 68.9% of the patients underwent at least a weekly session of ambulatory neurological rehabilitation therapy (average: 1.25 sessions per patient). These included physical therapy (61.3%), occupational therapy (23.8%), and cognitive rehabilitation (40.1%). We analyzed if patients with intermediate-severe dementia received more physical therapy rehabilitation than patients with mild dementia and we found no statistically significant difference (*χ* ^2^ (4) = 8.314, *p* = .08). More than 90% of the patients discontinued therapies during the pandemic. Also, this percentage did not differ significantly for persons in the three stages of dementia (*p* > .05)

Characteristics of participating caregivers of persons with AD or a related dementia are shown in Table 1, part II. The family was the primary provider of care in 65%, the average age and years of education of family caregivers were 56.21 ± 14.07 and 18.46 ± 6.84, respectively. Women accounted for 69.23% of family caregivers. Paid caregivers dedicated higher hours a day to caring for the patients with a CDR score > 2 compared to CDR score = 1.

Sixty percent of family members discontinued all visits to their loved ones, irrespectively of the severity of the dementia, in an attempt to limit the epidemic (*p* non-significant). However, only 28.6% of family caregivers chose to suspend services from paid caregivers. A statistically significant difference was observed in the percentages according to the severity of dementia, with the rate of non-suspension being higher in the more advanced stages (*χ* ^2^ (2) = 8.981, *p* = .011).

There was no difference in the level of burden before coronavirus pandemic for family caregivers of persons in the three stages of dementia (*p* > .05). However, the level of burden of the family caregiver after 4 weeks in quarantine was higher, especially for advanced stages of dementia (*χ* ^2^ (4) = 17.281, *p* = .002).

When we asked family member’s main concerns during the epidemic, we found some interesting and significant differences. Relatives of advance dementia subjects were more concern of the possibility of a sick leave of paid caregivers during the pandemic (*p* = .017), while relatives of subjects with mild dementia were mainly concerned of the risk of being contagious to the subjects when they assisted them with grocery supplies (*p* = .005).

## Discussion

This is the initial phase of this research on the effects of mandatory isolation on the care and caregiver stress of subjects with different stages of dementia living in the community.

The sample included a population of older adults with different stages of dementia, approximately 2/3 of the sample had moderate to severe dementia, and as expected, the most common cause of dementia in this older population was AD, followed by mixed AD. Approximately half of the subjects experienced increased anxiety during COVID isolation. Most patients received before the epidemic ambulatory rehabilitation services that included cognitive training, physical therapy, and occupational therapy, interesting subjects with more severe stages of the diseases received more therapies than subjects with milder disease. In most cases, outpatient rehabilitation services were suspended due to confinement independently of the severity of the disease.

As described in other studies done in Latin America, in our sample, caregiving usually relied on family members (Elnasseh *et al.*, 2016). Also, in concordance with published literature, we observed a disparity in gender distribution of family members caring of subjects with AD, with more women taking this role than men. As expected, subjects with a more severe stage of the disease had more hours of paid care due to functional dependence. One of our main findings was the high rate of discontinuation of family visits independently of the severity of the disease, probably related to fear of spreading the disease when visiting subjects. Another finding was that most families of subjects with severe dementia living in the community and not in long-term care facilities decided to continue with the assistance of a paid caregiver. This is likely due to the degree of dependence of this fragile population on caregivers.

Approximately half of the family caregivers reported that subjects with dementia experienced anxiety before the pandemic, irrespectively to the stage of the disease. With the enforced isolation due to COVID 19, this number increased. Loneliness, boredom, and lack of activities are known factors described in the literature to contribute to anxiety (Hwang *et al.*, 2020). Probably all these factors are playing an important role during quarantine. This finding is of concern, and extensive reports on the literature point the negative impact of anxiety on quality of life, functional dependence, and caregiver stress (Savva *et al.*, 2009).

Overall, COVID-19 confinement increased stress caregiver independently of the stage of the dementia, but those caring for severe dementia subjects had more COVID-related stress compared to caregivers assisting subjects with mild dementia. Main concerns of family members varied accordingly to the severity of the dementia. Advanced dementia relatives fear mostly of the absence of the paid caregiver, probably due to subject’s absolute dependence on caregivers for survival.

The main limitations of our study included the relatively small size of the sample and the lack of prospectively longitudinal follow-up. Also, there is a limitation related to our statistical analysis that must be considered: *p* values were not adjusted for multiple comparisons, and type I error rates might be high. We will replicate our study in a larger scale to analyze with more confidence if our results represent a real phenomenon and not just an inflated Type I error. Another limitation is that our main intention was to report in a preliminary way issues related to caregiver’s management during the pandemic and no validated instruments to measure variables such as burden of care or anxiety were tested. We will continue to follow this cohort of subjects prospectively to study the health consequences after the end of the isolation period and we will continue our work to test caregivers stress using validated scales.

Social distancing is effective as a global health policy for a disease that so far has no vaccination or approved treatment. In Argentina, main efforts of the authorities had been to identity, isolate, and treat sick individuals and social services and help was mainly assigned to the care of symptomatic and severely ill patients and families. In older adults with dementia, we observed a rise in clinically relevant complications due to social isolation and we fear that this population may suffer more functionally dependence, dementia progression, and worsening of their quality of life. Our results showed that caregivers are already grappling with rising stress levels caused by the pandemic. We urge social services and governments to address specific interventions for the population of subjects with dementia and their relatives. A partnership between departments of public health, care workers, and families must be planned to guarantee continuity of care. A post-COVID-19 reality must be considered to ameliorate the real impact of mandatory social isolation in vulnerable populations with cognitive decline.
